# Comparative Analysis of Mesocotyl Elongation Ability among Maize Inbred Lines

**DOI:** 10.3390/ijms252212437

**Published:** 2024-11-19

**Authors:** Daxing Wen, Xiaoyu Tian, Chenglai Wu, Chunqing Zhang

**Affiliations:** Agronomy College, Shandong Agricultural University, Tai’an 271018, China; dxwen@sdau.edu.cn (D.W.); qq1842362293@126.com (X.T.); clwu@sdau.edu.cn (C.W.)

**Keywords:** mesocotyl, maize, inbred line, transcriptome

## Abstract

Mesocotyl plays a key role in the seedling emergence of maize; however, the mechanism of mesocotyl elongation is still unclear. Moreover, different maize inbred lines and cultivars have varied mesocotyl lengths positively correlated with deep sowing tolerance. In this study, we selected one inbred line with long mesocotyl (LM) and two maize inbred lines with short mesocotyl (SM1 and SM2) from more than 400 maize inbred lines. The mesocotyl length of the LM line was about three-fold longer than those of the SM1 and SM2 lines. Microstructure observation showed that the reason for short mesocotyl in the SM1 and SM2 lines was few cell numbers and short cell length, respectively. Subsequently, we used RNA-seq to investigate the mechanism of mesocotyl elongation by regulating cell number and cell length at the transcriptome level. Compared with the LM line, the SM1 line displayed stronger downregulation of *Cytochrome P450* and *peroxidase* genes than the SM2 line. Moreover, plant hormone signal transduction plays a vital role in mesocotyl elongation. Taken together, we propose a model for mesocotyl elongation of maize inbred lines with different cell lengths and cell numbers, which provide new insights into mesocotyl elongation in maize.

## 1. Introduction

Mesocotyl is an important organ between the coleoptile node and the basal part of the seminal root in Gramineae, which plays a crucial role in the seedling emergence of maize, sorghum, and rice [1,2]. Arabidopsis mainly depends on hypocotyl elongation during seedling emergence [3]. The process of seedling emergence involves transitioning from skotomorphogenesis to photomorphogenesis [4]. Mesocotyl elongation usually occurs in the skotomorphogenesis stage in the soil layer. Numerous studies have revealed the mechanism of hypocotyl elongation; however, the mechanism of mesocotyl elongation is still unclear.

Deep sowing, a method commonly used in arid soils, can significantly induce mesocotyl elongation of maize with deep sowing tolerance [5]. Proper deep sowing can effectively alleviate the damage to maize seedlings caused by drought and low-temperature stress, which is an important measure to avoid drought and cold at the seedling stage [6]. Maize is usually sown to a depth of about 5 cm, while deep sowing can stimulate seed using water from the deep soil to germinate [5]. The mesocotyl length of maize materials with deep sowing tolerance is much longer than that of maize materials sensitive to deep sowing, and the mesocotyl length is significantly positively correlated with deep sowing tolerance [2]. Many studies have shown that mesocotyl elongation is the main reason for maize deep sowing tolerance [7]. Moreover, coleoptile elongation is also a driving force of maize seedlings under deep sowing, but the mesocotyl is more critical than the coleoptile elongation [8,9].

Different maize inbred lines and cultivars have varied mesocotyl lengths because of the genetic diversity of maize. Seedling emergence is fast and uniform in maize materials with long mesocotyl. Under drought stress during the sowing period of maize, appropriately increasing the sowing depth can help the seeds absorb more soil water and promote seed germination and seedling establishment [5]. The mesocotyl length is closely related to the seedling emergence rate [7]. However, maize materials with short mesocotyl are sensitive to deep sowing, failing to break through the soil layer and decreasing seedling emergence rate [10]. Therefore, revealing the mechanism of mesocotyl elongation is of great importance for ensuring rapid and uniform seedling emergence.

Mesocotyl length is a quantitative trait controlled by multiple genes. Many mesocotyl length or deep sowing tolerance-related QTL loci are mapped using GWAS and QTL in maize and rice [1,2,7,8,11]. Under 10 cm and 20 cm sowing depth, deep sowing tolerance-related QTL loci are mapped to chromosomes 1, 3, 4, 6, 7, and 10 in maize by the F_2_ population constructed with maize inbred lines 3681-4 and X178 [2]. Moreover, 33 QTLs involved in deep sowing tolerance of maize are identified by composite interval mapping, and 50 candidate genes are predicted using RNA-seq data [6]. Using a DH population constructed by B73 and Mo17, a previous study identified 13 QTLs related to mesocotyl length, explaining 2.5–7.8% of phenotypic variance [8]. The QTL regions that have been reported are extensive, indicating that fine mapping with larger populations and more precise and accurate phenotypes is necessary [12]. Many mesocotyl length or deep sowing tolerance-related QTL loci have been identified; however, gene cloning and functional verification involved in maize mesocotyl elongation are still very limited. In particular, the key genes determining mesocotyl length in maize are unknown. It might be possible to mine some genes related to the regulation of mesocotyl elongation based on multi-omics techniques, such as transcriptome and proteome analysis.

Besides genetic factors, mesocotyl elongation is also influenced by environmental conditions and plant hormones [4,13,14]. Mesocotyl elongation is inhibited under light and promoted significantly in darkness [15]. Polyamine oxidase (PAO) activity in maize mesocotyl increases under light, leading to cell wall hardening and inhibiting mesocotyl elongation [16]. The optimum temperature promotes mesocotyl elongation, but neither high nor low temperature is conducive to mesocotyl elongation [12]. Mesocotyl tissue is more vulnerable to low-temperature stress than other tissues during maize seed germination. Therefore, mesocotyl tissue can be used to evaluate the cold tolerance of maize [17]. Mesocotyl elongation is regulated by many plant hormones, such as Indole-3-acetic acid (IAA), cytokinin (CK), gibberellin (GA), abscisic acid (ABA), ethylene (ETH), brassinosteroid (BR), strigolactones (SLs), and jasmonic acid (JA) [9,10]. Plant hormones generally control mesocotyl elongation by regulating cell division or elongation [18]. Various plant hormones regulate rice mesocotyl elongation through complex regulatory pathways [12]. Maize mesocotyl elongation requires both IAA and BR, and BR inhibitors weaken mesocotyl elongation [13,19,20]. The network of plant hormones regulating maize mesocotyl elongation remains ambiguous.

This study aimed to investigate the differences in mesocotyl elongation among maize inbred lines with various mesocotyl lengths at the transcriptome level. We first selected one inbred line with long mesocotyl (LM) and two maize inbred lines with short mesocotyl (SM1 and SM2) from more than 400 maize inbred lines for transcriptome analysis. The reason for short mesocotyl in SM1 and SM2 was a few cell numbers and short cell lengths, respectively. Subsequently, we used RNA-seq to analyze the samples at germination 5 d under dark conditions when mesocotyl grew rapidly. We propose a possible network of mesocotyl elongation of maize inbred lines with different cell lengths and cell numbers, which provide new insights into mesocotyl elongation in maize.

## 2. Results

### 2.1. Few Cell Numbers and Short Cell Length Are the Key Factors of Short Mesocotyl Length in Maize

**Figure 1:**
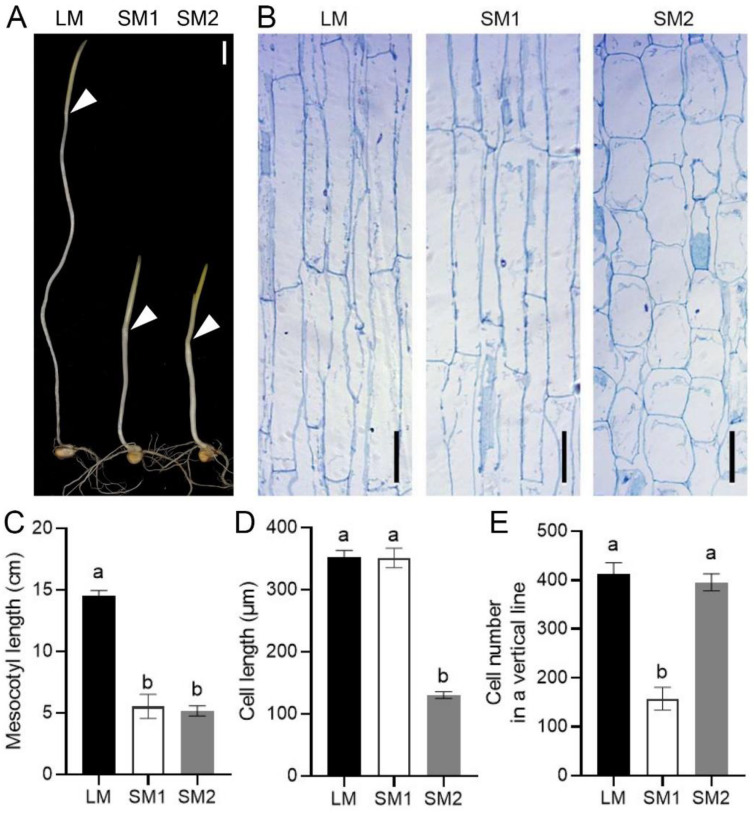
Phenotypes of three maize inbred lines: (**A**) Mesocotyl at germination 7 d under dark conditions, scale bar represents 1 cm, arrows indicate the coleoptile nodes; (**B**) Images of the vertical section of the center of mesocotyl, scale bars represent 100 μm; (**C**) Mesocotyl length; (**D**) Mesocotyl cell length; (**E**) Mesocotyl cell number in a vertical line. LM is a maize inbred line with long cells and many cell numbers in mesocotyl. SM1 is a maize inbred line with long cells and few cell numbers in mesocotyl. SM2: a maize inbred line with short cell and many cell numbers in mesocotyl. Error bars display the standard deviation for three replicates. Different letters show significant differences among treatments (*p*-value < 0.05).

Light can inhibit mesocotyl elongation. To investigate the potential of mesocotyl elongation, we detected the mesocotyl length of more than 400 maize inbred lines after germination 7 d under dark conditions. The results showed that mesocotyl length significantly differed among these inbred lines at germination 7 d (Figure 1A). Paraffin sectioning displayed short mesocotyl length due to few cell numbers or short cell lengths (Figure 1B). In exploring the reasons for short mesocotyl due to few cell numbers, we wanted to exclude or decrease the effects of the cell length. In contrast, the effects of the cell number were eliminated or reduced when we explored the reasons for short mesocotyl due to short cell lengths. Therefore, screening suitable materials was difficult and was needed to measure the numerous materials. Subsequently, we selected one inbred line with long mesocotyl (LM) and two maize inbred lines with short mesocotyl (SM1 and SM2) for transcriptome analysis. The mesocotyl length of the LM line was about three-fold longer than those of the SM1 and SM2 lines (Figure 1C). The SM1 line had a similar cell length compared with the LM line, but the cell number of the SM1 line was remarkably fewer than that in the LM line (Figure 1D,E). There was no significant difference in cell number between the SM2 and LM lines. However, the SM2 line displayed a notably shorter cell length than the LM line. The cells of mesocotyl in the SM2 line were thicker than the LM and SM1 lines (Figure 1B), but the length of mesocotyl is more important for seedlings breaking through the soil layer than the thickness of mesocotyl. Thus, we did not focus on the thickness of mesocotyl. In the subsequent study, we used the SM1 line compared with the LM line (SM1vsLM) and the SM2 line compared with the LM line (SM2vsLM) to investigate the mechanism of mesocotyl elongation by regulating cell number and cell length, respectively.

### 2.2. Transcriptome Analysis of Mesocotyl Elongation

To explore the regulation network of mesocotyl elongation of maize inbred lines with different cell lengths and cell numbers, we performed transcriptome analysis on samples at germination 5 d under dark conditions when mesocotyl grew rapidly. Mesocotyl samples of three maize inbred lines (LM, SM1, and SM2) with three biological replicates were used for RNA-seq, which generated about 61.45–66.96 million raw reads for each sample (Supplementary Table S1). Then, adapters and sequences with low-quality regions were removed, with nearly 57.42–64.67 million clean reads remaining. About 49.24–58.28 million clean reads were mapped to the maize genome. The clean reads included 83.75%–88.73% uniquely mapped reads and 1.90%–2.09% multiple mapped reads. Subsequently, the DESeq2 R package (1.20.0) was used to identify differentially expressed genes (DEGs) by using adjusted *p* <0.05 and |log_2_Foldchange| ≥ 1 as the cutoff. The results showed that 3587 genes were notably upregulated and 4644 genes were significantly downregulated in SM1vsLM. Moreover, 4338 genes were remarkably upregulated and 3690 genes were markedly downregulated in SM2vsLM.

### 2.3. Many Cytochrome P450 and Peroxidase-Related Genes Were Downregulated in the Short Mesocotyl Lines Compared with the Long Mesocotyl Line

**Figure 2:**
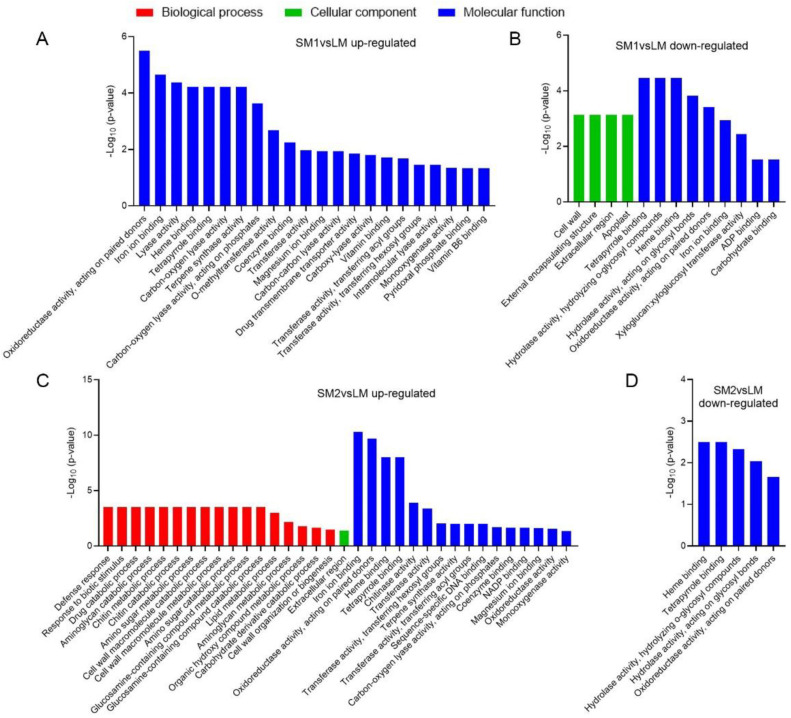
Significantly enriched Gene Ontology (GO) terms: (**A**) GO terms for the upregulated genes in SM1vsLM. (**B**) GO terms for the downregulated genes in SM1vsLM. (**C**) GO terms for the upregulated genes in SM2vsLM. (**D**) GO terms for the downregulated genes in SM2vsLM. GO terms were sorted based on *p*-values. LM is a maize inbred line with long cells and many cell numbers in mesocotyl. SM1 is a maize inbred line with long cells and few cell numbers in mesocotyl. SM2: a maize inbred line with short cell and many cell numbers in mesocotyl.

**Figure 3:**
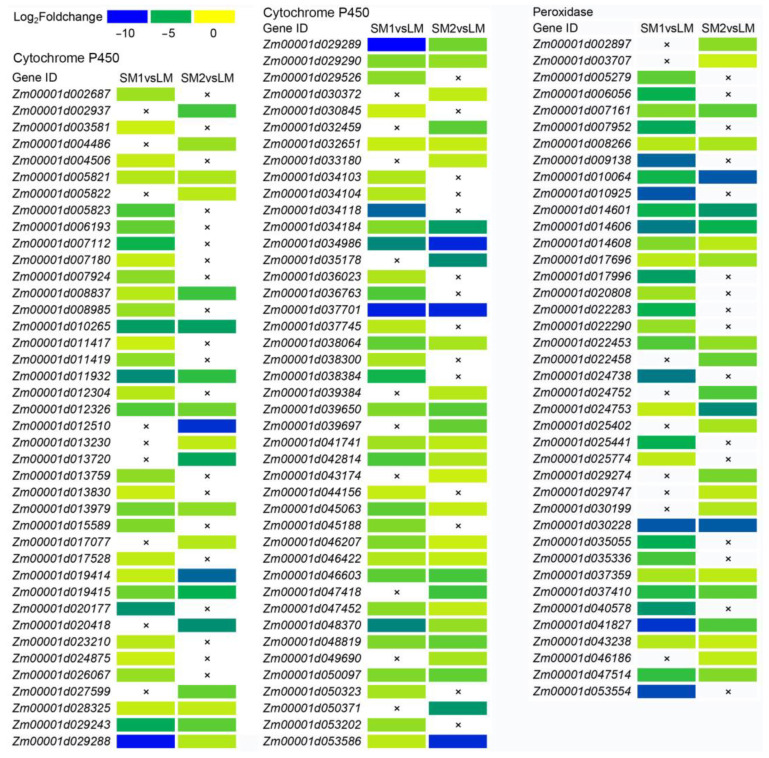
Heat map of selected DEGs annotated Cytochrome P450 and peroxidase from GO terms for the downregulated genes in SM1vsLM and SM2vsLM. LM is a maize inbred line with long cells and many cell numbers in mesocotyl. SM1 is a maize inbred line with long cells and few cell numbers in mesocotyl. SM2: a maize inbred line with short cell and many cell numbers in mesocotyl. The × represents that it is not a DEG in the comparison.

To further understand the function of these DEGs, we performed a Gene Ontology (GO) term enrichment analysis in two comparisons of SM1vsLM and SM2vsLM. The results showed that all the significantly enriched GO terms of the upregulated DEGs in SM1vsLM belonged to the molecular function group, in which the most significantly enriched GO term was oxidoreductase activity (acting on paired donors, GO: 0016705, *p* = 3.20 × 10^−6^) (Figure 2A). For the downregulated DEGs in SM1vsLM, there were four significantly enriched GO terms in the cellular component group and nine significantly enriched GO terms in the molecular function group (Figure 2B). Cell wall (GO: 0005618, *p* = 7.36 × 10^−4^) belonged to the cellular component group and included 20 downregulated DEGs that were annotated xyloglucan endotransglucosylase/hydrolase protein, pectinesterase, or pectinesterase inhibitor (Table 1). For the upregulated DEGs in SM2vsLM, there were 17 significantly enriched GO terms in the biological process group and 16 significantly enriched GO terms in the molecular function group, with only one significantly enriched GO term in the cellular component group (Figure 2C). Among them, the most significantly enriched GO term was iron ion binding (GO: 0005506, *p* = 4.97 × 10^−11^) in the molecular function group. All the significantly enriched GO terms of the downregulated DEGs in SM2vsLM belonged to the molecular function group (Figure 2D). To understand which GO terms might decrease mesocotyl length, we focus on the downregulated GO terms in SM1vsLM and SM2vsLM. Interestingly, the top three significantly enriched GO terms in SM1vsLM were similar to those in SM2vsLM (Figure 2B,D), that is tetrapyrrole binding (GO: 0046906), hydrolase activity (GO: 0004553) and heme binding (GO: 0020037). Moreover, the DEGs in tetrapyrrole binding (GO: 0046906) were the same as heme binding (GO: 0020037) in SM2vsLM, and tetrapyrrole binding (GO: 0046906) had one more DEG than heme binding (GO: 0020037) in SM1vsLM. In the two GO terms, approximately 60% of DEGs were annotated Cytochrome P450, and about 30% of DEGs were annotated peroxidase. Some DEGs were exhibited in both SM1vsLM and SM2vsLM, while other DEGs were exhibited in only SM1vsLM or SM2vsLM (Figure 3). Hydrolase activity (GO: 0004553) displayed various hydrolase-related genes, including some cell wall metabolism-related genes.

### 2.4. Plant Hormone Signal Transduction-Related Genes Are Involved in Regulating the Length and Number of Mesocotyl Cell

**Figure 4:**
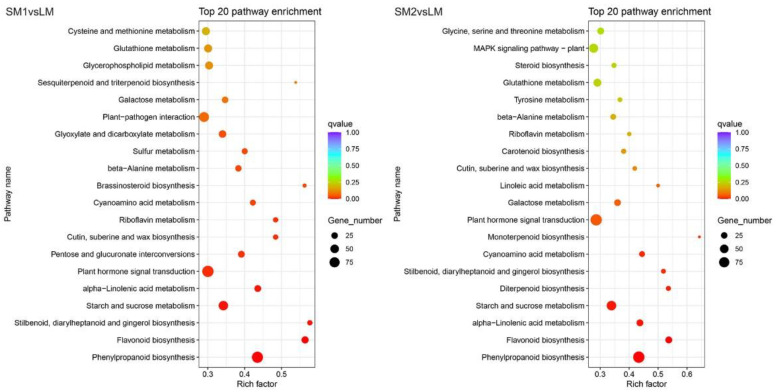
Top 20 Kyoto Encyclopedia of Genes and Genomes (KEGGs) pathways in SM1vsLM and SM2vsLM. LM is a maize inbred line with long cells and many cell numbers in mesocotyl. SM1 is a maize inbred line with long cells and few cell numbers in mesocotyl. SM2: a maize inbred line with short cell and many cell numbers in mesocotyl.

**Figure 5:**
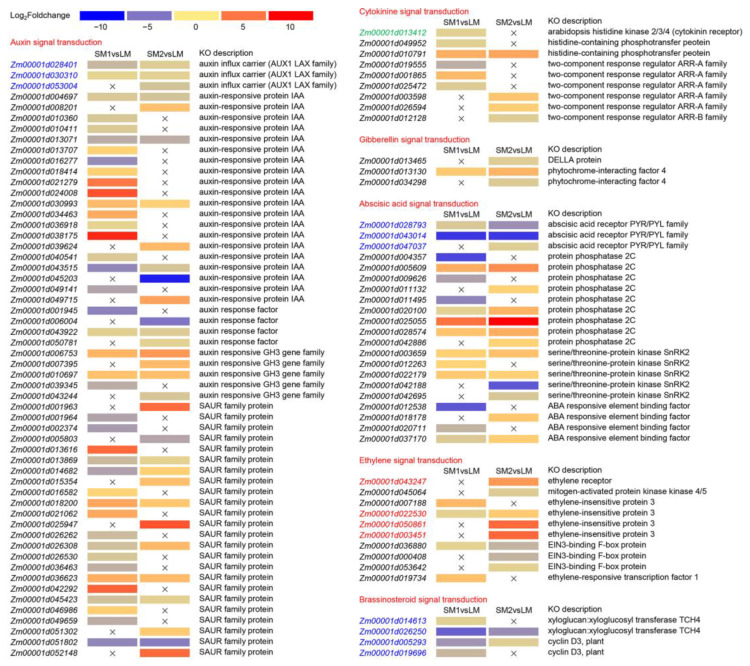
Heat map of selected DEGs from the plant hormone signal transduction pathway in SM1vsLM and SM2vsLM. LM is a maize inbred line with long cells and many cell numbers in mesocotyl. SM1 is a maize inbred line with long cells and few cell numbers in mesocotyl. SM2: a maize inbred line with short cell and many cell numbers in mesocotyl. The × represents that it is not a DEG in the comparison.

To identify the metabolic pathways involved in mesocotyl elongation, we further analyzed the Kyoto Encyclopedia of Genes and Genomes (KEGG) enrichment pathways. The top two pathways were phenylpropanoid biosynthesis and flavonoid biosynthesis in both SM1vsLM and SM2vsLM, which might play important roles in mesocotyl elongation (Figure 4). Moreover, stilbenoid, diarylheptanoid, gingerol biosynthesis, starch and sucrose metabolism, alpha-linolenic acid metabolism, and plant hormone signal transduction were also significantly enriched in the two comparisons. Numerous studies have reported that many plant hormone-related genes participate in Arabidopsis hypocotyl elongation and mesocotyl elongation in rice and maize [1,3,9,10]. Therefore, we further analyzed the DEGs in the plant hormone signal transduction pathway. The results showed that approximately half of the DEGs in the plant hormone signal transduction pathway were involved in auxin signal transduction, followed by abscisic acid, ethylene, cytokinin, brassinosteroid, and gibberellin signal transduction in SM1vsLM and SM2vsLM (Figure 5). In auxin signal transduction pathway, three auxin influx carrier (AUX1 LAX family) genes (*Zm00001d028401*, *Zm00001d030310*, and *Zm00001d053004*) were downregulated in SM2vsLM, in which *Zm00001d028401* and *Zm00001d030310* were also downregulated in SM1vsLM. In abscisic acid signal transduction, three abscisic acid receptor PYR/PYL family genes (*Zm00001d028793*, *Zm00001d043014*, and *Zm00001d047037*) were downregulated in SM2vsLM, in which *Zm00001d028793* and *Zm00001d043014* were also downregulated in SM1vsLM. All four DEGs (*Zm00001d014613*, *Zm00001d026250*, *Zm00001d005293*, and *Zm00001d019696*) in brassinosteroid signal transduction pathway were downregulated in both SM1vsLM and SM2vsLM. However, one DEG (*Zm00001d013412*) annotated Arabidopsis histidine kinase 2/3/4 (cytokinin receptor) in cytokinine signal transduction was downregulated only in SM1vsLM. One DEG (*Zm00001d043247*) annotated ethylene receptor, and three DEGs (*Zm00001d022530*, *Zm00001d050861*, and *Zm00001d003451*) annotated ethylene-insensitive protein. Three were upregulated only in SM2vsLM.

### 2.5. Validation of RNA-Seq Data

**Figure 6:**
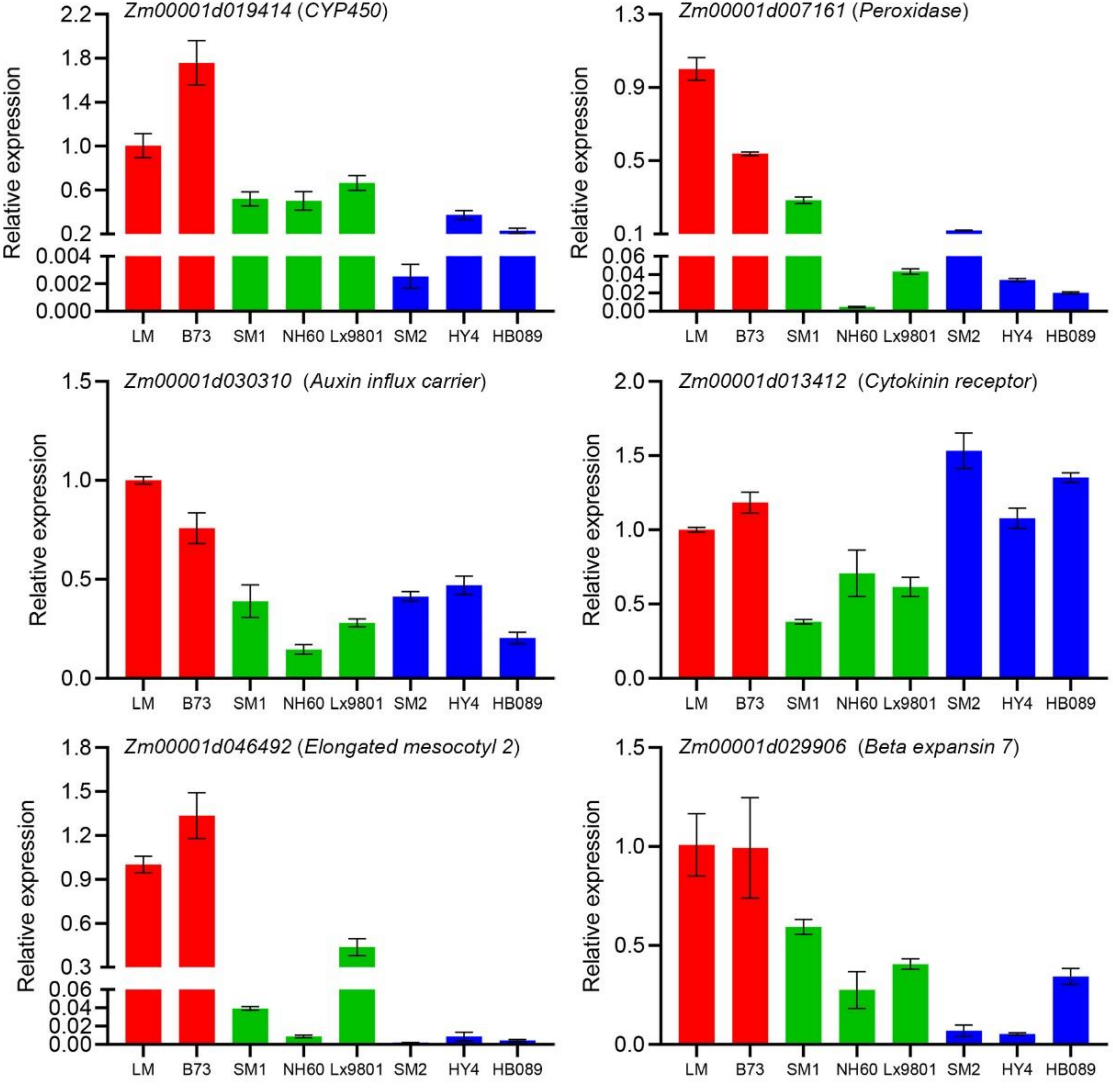
Validation of the expression levels of DEGs in more maize inbred lines using qRT-PCR. Red bars represent the maize inbred lines with long mesocotyl. Green bars represent the maize inbred lines with long cells and few cell numbers in mesocotyl. Blue bars represent the maize inbred lines with short cells and many cell numbers in mesocotyl. Error bars represent the standard deviation for three replicates.

To validate the DEGs identified using RNA-seq, we randomly selected *Zm00001d019414* (*CYP450*) and *Zm00001d007161* (*Peroxidase*) from Figure 3, *Zm00001d030310* (*Auxin influx carrier*) and *Zm00001d013412* (*Cytokinin receptor*) from Figure 5, and *Zm00001d046492* (*Elongated mesocotyl 2*) and *Zm00001d029906* (*Beta expansin 7*) from other DEGs to perform quantitative real-time PCR (qRT-PCR) analyses. The results displayed that the expression patterns of these genes in the qRT-PCR assays were similar to those transcript abundance changes identified by transcriptome analyses (Supplementary Figure S1). Moreover, we selected another maize inbred line with long mesocotyl (B73), two maize inbred lines with long cells and few cell numbers in mesocotyl (NH60 and Lx9801), and two maize inbred lines with short cell and many cell numbers in mesocotyl (HY4 and HB089) to detect the expression level of the above six genes. NH60 and Lx9801 had relatively short mesocotyl mainly due to few cell numbers; however, the cell lengths might also be different compared with LM and B73 (Supplementary Figure S2). Although HY4 and HB089 showed short mesocotyl mainly due to short cells, the cell numbers might also be different compared with LM and B73 (Supplementary Figure S2). The qRT-PCR results showed that the newly selected maize inbred lines displayed similar expression patterns with the maize inbred lines in transcriptome analyses (Figure 6). The above results indicated that the RNA-seq data were reliable.

## 3. Discussion

Under dark conditions, the growth of maize mesocotyl shows a slow-fast-slow trend, along with significant changes in the contents of auxin, cellulose, and POD activity [21]. The POD activity of mesocotyl is notably increased in light than in darkness [15]. Under the condition of no control of light, skotomorphogenesis, and photomorphogenesis affected the mesocotyl elongation together. Therefore, the mechanism of mesocotyl elongation under no control of light was very complicated. To eliminate the influence of light on mesocotyl elongation, we evaluated maize mesocotyl length via germination under dark conditions to explore the mechanism of mesocotyl elongation in maize. Moreover, we found many DEGs annotated Cytochrome P450 and peroxidase were downregulated in both SM1vsLM and SM2vsLM (Figure 3). Cytochrome P450 family genes in plants are involved in various physiological processes, such as plant metabolism, stress responses, phytohormones, and signaling molecules [22,23]. POD is an essential enzyme in lignin metabolism related to maize mesocotyl elongation [24]. Therefore, *Cytochrome P450* and *peroxidase* genes might play important roles in mesocotyl elongation in maize.

A previous study identified three xyloglucan endotransglucosylase/hydrolase genes regulating mesocotyl elongation in sorghum based on transcriptome analysis [25]. In this study, we found that 13 DEGs annotated xyloglucan endotransglucosylase/hydrolases were downregulated in SM1vsLM by GO enrichment analysis. These results indicated that xyloglucan endotransglucosylase/hydrolases might play important roles in the mesocotyl elongation of maize and sorghum.

Many plant hormones regulate mesocotyl elongation in maize and rice and hypocotyl elongation in Arabidopsis. Plant hormones affect mesocotyl length by regulating cell number and cell length [12]. The primary source of IAA in mesocotyl is the coleoptile unit (including the primary leaf and coleoptile segment), and more than 50% of IAA comes from the coleoptile tip [26]. The IAA content in the epidermis irradiated by red light is lower than that of the control in darkness [27,28]. The growth rate of mesocotyl at 20 cm depth is 1.5–2 times that at 2 cm depth, mainly due to the regulation of the rapid elongation of mesocotyl by increasing IAA synthesis and transport [29]. In this study, different maize inbred lines showed significant differences in mesocotyl length, and the expression levels of three *AUX1* genes encoding auxin influx carrier in the auxin signal transduction pathway were downregulated in short mesocotyl maize inbred lines, indicating that auxin plays a vital role in the rapid elongation of mesocotyl.

ABA promotes the growth of rice mesocotyl by prolonging the cell division activity of meristem, and fluridone (FLU, an inhibitor of ABA biosynthesis) inhibits mesocotyl elongation [30]. BR can promote mesocotyl elongation by inhibiting the phosphorylation of U-type cyclin CYC U2 by OsGSK2 [1]. Moreover, 2.0 mg/L exogenous 24-epibrassinolide significantly increased the mesocotyl length of maize [9]. In this study, the expression of ABA receptor *PYR/PYL* family genes and *TCH4* and *CYCD3* genes in the BR signal transduction pathway were downregulated in short mesocotyl maize inbred lines, indicating that ABA and BR promote mesocotyl elongation, which is consistent with previous studies.

The antagonism of CK and SLs regulates the elongation of rice mesocotyl in the dark, and *d10-1* and *d14-1* mutants are more sensitive to CK than the wild type [31]. In this study, the cytokinin receptor *CRE1* gene was downregulated only in maize inbred line SM1 with few mesocotyl cell numbers, indicating that cytokinin signaling regulates mesocotyl cell number.

Ethephon and coronatine can decrease mesocotyl length by inhibiting cell elongation in maize [32]. Ethylene-insensitive protein 3 (EIN3) in the ethylene signal transduction pathway slows down the elongation of hypocotyl cells in Arabidopsis by activating the ERF1 pathway [3]. In this study, the expression levels of ethylene receptor *ETR* and *EIN3* genes in the ethylene signal transduction pathway were only upregulated in maize inbred line SM2 with short mesocotyl cell length, indicating that they might play an important role in inhibiting mesocotyl elongation in maize, similar to the results in Arabidopsis.

Maize mesocotyl elongation is more sensitive to GA_3_ under 20 cm sowing depth than under 2 cm sowing depth [33]. Moreover, exogenous GA promotes mesocotyl elongation under deep sowing conditions [34]. Gene chip analysis and exogenous GA processing showed that *ZmMYB59* responded to deep sowing through the GA signaling pathway in maize [35]. Dynamic transcriptome and plant hormone analysis of rice mesocotyl elongation in response to light showed that light reduced the contents of IAA and GA_3_ and increased JA levels to inhibit mesocotyl elongation [36]. In this study, compared with the DEGs in other plant hormone signal transduction pathways, maize inbred lines with different mesocotyl lengths had the fewest DEGs in GA signal transduction pathways, and the degree of upregulation and downregulation was also relatively low. The results indicate that GA signal transduction might not be the main reason for varied mesocotyl length in this study.

**Figure 7:**
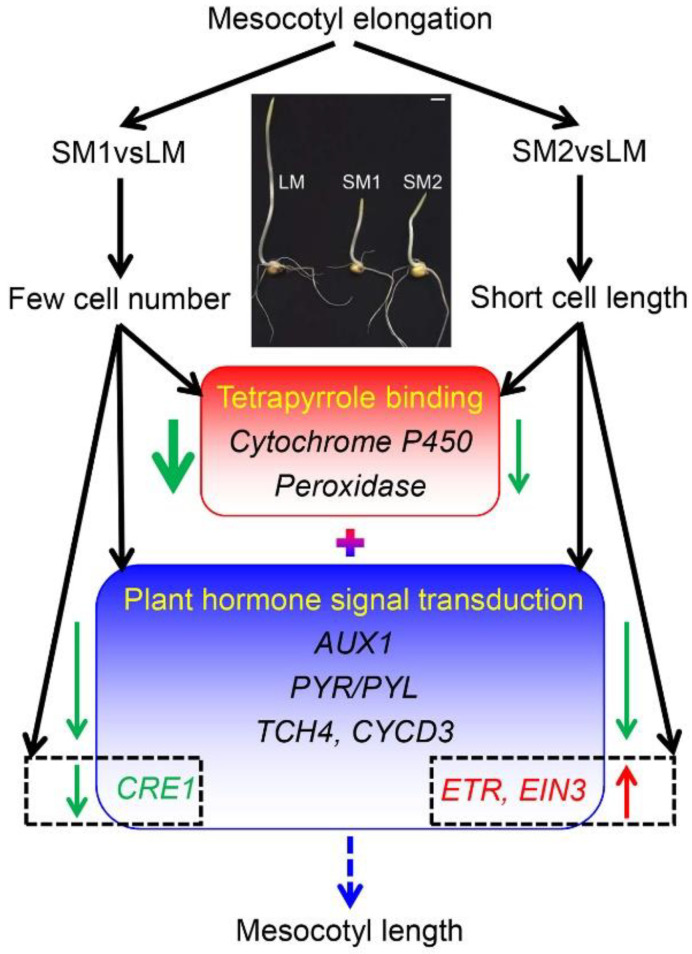
A possible network of mesocotyl elongation of maize inbred lines with different cell lengths and cell numbers. The red and green arrows indicate upregulated and downregulated genes, respectively. The thickened green arrow means stronger downregulation. LM is a maize inbred line with long cells and many cell numbers in mesocotyl. SM1 is a maize inbred line with long cells and few cell numbers in mesocotyl. SM2: a maize inbred line with short cell and many cell numbers in mesocotyl. *AUX1: auxin influx carrier (AUX1 LAX family). PYR/PYL: abscisic acid receptor PYR/PYL family. TCH4: xyloglucan:xyloglucosyl transferase TCH4. CYCD3: cyclin D3. CRE1: cytokinin receptor. ETR: ethylene receptor. EIN3: ethylene-insensitive protein 3*.

Taken together, we propose a possible network of mesocotyl elongation of maize inbred lines with different cell lengths and cell numbers (Figure 7). Compared with the maize inbred line with long mesocotyl, the maize inbred line with few cell number of mesocotyl displayed stronger downregulation of *Cytochrome P450* and *peroxidase* genes than the maize inbred line with short cell length of mesocotyl. Moreover, plant hormone signal transduction plays important roles in mesocotyl elongation, in which *AUX1*, *PRY/PRL*, *TCH4*, and *CYCD3* genes involved in auxin, abscisic acid, and brassinosteroid signal transduction are downregulated in the maize inbred lines with few cell number or short cell length of mesocotyl. Notably, *CRE1* in cytokinin signal transduction is downregulated only in the maize inbred line with few cell numbers of mesocotyl. However, *ETR* and *EIN3* genes related to ethylene signal transduction are upregulated only in the maize inbred line with a short cell length of mesocotyl. The expression levels of the above genes might determine mesocotyl length.

## 4. Materials and Methods

### 4.1. Materials

To investigate the differences in mesocotyl elongation among maize inbred lines with long and short mesocotyl, we selected three maize inbred lines (LM, SM1, and SM2) with different mesocotyl lengths from more than 400 maize inbred lines. Moreover, another maize inbred line with long mesocotyl (B73), two maize inbred lines with long cells and few cell numbers in mesocotyl (NH60 and Lx9801), and two maize inbred lines with short cells and many cell numbers in mesocotyl (HY4 and HB089) to validate RNA-seq data. These maize inbred lines were grown at the experimental station of Shandong Agricultural University (36°90′ N and 117°90′ E, Tai’an City, Shandong Province, China). The newly harvested seeds were used for subsequent experiments.

### 4.2. Measurement of Mesocotyl Length

Maize seeds were sown in a sprouting bed consisting of silica sand with 60% saturation moisture content at 1 cm sowing depth in a germination box. Then, the seeds were kept at 25 ± 1 °C in darkness for seven days. After washing silica sand from maize seedlings, mesocotyl length was measured by a ruler. The mean of about 30 mesocotyl length represented the mesocotyl length per replicate. Each maize inbred line included three replicates.

### 4.3. Measurement of Cell Length and Cell Number of Mesocotyl

Cell length and cell number in a vertical line of mesocotyl were measured by microstructure observation according to a previous study with minor modifications [37]. Approximately 1 cm of the middle part of mesocotyl was immersed in 50% formalin–acetic acid–alcohol (FAA) fixative. Paraffin sectioning and histological staining were performed by a previous study [37]. The longitudinal sections of the middle part of mesocotyl were observed by a microscope. Cell length was measured from the mean length of 10 randomly selected cells per sample. In the longitudinal section of mesocotyl, meristematic cells, rapidly growing cells, and mature cells are located in the 1.0 mm, 3.0 mm, and 5.0 mm zones below the mesocotyl node, respectively [38]. In this study, the apical meristematic part and the rapid elongation part are much shorter (about 0.5 cm) compared to the middle elongation part and the lower mature part at germination 7 d under dark conditions. Thus, the cell number in a vertical line of mesocotyl was calculated by dividing the mesocotyl length into cell length.

### 4.4. RNA-Seq and Transcriptome Analysis

Mesocotyl samples from 30 seedlings at germination 5 d under dark conditions were pooled together as one biological replicate. Each treatment included three biological replicates. Mesocotyl samples were frozen in liquid nitrogen and then kept at −80 °C until RNA extraction. Frozen samples were ground by using a ball mill. The ground samples (approximately 0.1 g) were used for total RNA extraction by the RNA extraction kit DP441 (Tiangen, Beijing, China). RNA integrity and concentration were examined as previously described [39]. RNA-seq library construction and sequencing were conducted according to a previous study [40]. Raw reads produced from RNA-seq were preprocessed to obtain clean reads, and then they were mapped to the maize reference genome sequence (B73 v4, ftp.ensemblgenomes.org/pub/plants/release-47/fasta/zea_mays/dna/ accessed on 10 October 2024) by using HISAT2. Feature Counts (v1.5.0-p3) were used to count the number of reads mapped to each gene. Differential expression analysis of two groups was analyzed by using the DESeq2 R package (1.20.0) [41]. Then, the *p* values were adjusted according to the Benjamini and Hochberg algorithm. Differentially expressed genes (DEGs) were identified by using adjusted *p* < 0.05 and |log_2_Foldchange| ≥ 1 as the cutoff. Gene Ontology (GO) enrichment analysis and Kyoto Encyclopedia of Genes and Genomes (KEGG) pathways enrichment analysis were performed as previously described [40].

### 4.5. qRT-PCR

The qRT-PCR assays were performed according to a previous study [39]. Primer 6 software was used to design gene-specific primers, which are listed in Supplementary Table S2. A PrimeScript RT reagent kit (Takara, Dalian, China) was applied to synthesize cDNA. All qRT-PCR assays were repeated at least three times. The maize *Actin* gene (*Zm00001d010159*) was used as an internal control to normalize the expression levels of the selected genes. The primers of the *Actin* gene came from a previous study [42]. The relative expression levels of genes were calculated by using the 2^−ΔΔCt^ method [43].

### 4.6. Statistical Analysis

We used SPSS 19.0 software (SPSS, Chicago, IL, USA) to perform statistical analysis.

## 5. Conclusions

The reason for short mesocotyl in the SM1 and SM2 lines was a few cell numbers and short cell lengths, respectively. Transcriptome analysis revealed that *Cytochrome P450*, *peroxidase* genes, and plant hormone signal transduction pathway play important roles in mesocotyl elongation. Taken together, we propose a model of mesocotyl elongation among maize inbred lines with different cell lengths and cell numbers, which provide new insights into mesocotyl elongation in maize.

## Figures and Tables

**Table 1:** DEGs in the cell wall GO term in SM1vsLM.

Gene ID	Gene Annotation	log2FoldChange	*p*-Value
*Zm00001d044775*	Xyloglucan endotransglucosylase/hydrolase protein 3	−9.42	7.18 × 10^−14^
*Zm00001d026250*	Xyloglucan endotransglucosylase/hydrolase protein 24	−5.92	4.22 × 10^−4^
*Zm00001d051526*	Probable xyloglucan endotransglucosylase/hydrolase protein 30	−5.65	8.86 × 10^−4^
*Zm00001d026251*	Probable xyloglucan endotransglucosylase/hydrolase protein 16	−4.87	1.52 × 10^−2^
*Zm00001d024378*	Xyloglucan endotransglucosylase/hydrolase 2	−4.77	2.05 × 10^−11^
*Zm00001d050201*	Probable xyloglucan endotransglucosylase/hydrolase protein 25	−3.91	3.85 × 10^−4^
*Zm00001d002409*	Probable xyloglucan endotransglucosylase/hydrolase protein 16	−3.80	6.49 × 10^−3^
*Zm00001d009899*	Probable pectinesterase/pectinesterase inhibitor 41	−3.54	4.49 × 10^−10^
*Zm00001d022104*	Pectinesterase QRT1	−3.47	2.28 × 10^−8^
*Zm00001d024392*	Probable xyloglucan endotransglucosylase/hydrolase protein 25	−3.07	1.88 × 10^−3^
*Zm00001d053961*	Probable xyloglucan endotransglucosylase/hydrolase protein 30	−3.02	6.85 × 10^−3^
*Zm00001d032992*	Pectinesterase 31	−2.27	1.24 × 10^−49^
*Zm00001d047970*	Probable xyloglucan endotransglucosylase/hydrolase protein 28	−2.01	2.98 × 10^−22^
*Zm00001d045048*	Probable pectinesterase/pectinesterase inhibitor 12	−1.83	3.38 × 10^−53^
*Zm00001d002412*	Probable xyloglucan endotransglucosylase/hydrolase protein 25	−1.69	2.74 × 10^−3^
*Zm00001d042624*	Probable pectinesterase/pectinesterase inhibitor 51	−1.39	3.49 × 10^−36^
*Zm00001d012766*	Probable pectinesterase 53	−1.31	7.44 × 10^−9^
*Zm00001d042625*	Probable pectinesterase/pectinesterase inhibitor 51	−1.22	7.04 × 10^−28^
*Zm00001d014613*	Xyloglucan endotransglucosylase/hydrolase protein 22	−1.17	1.43 × 10^−21^
*Zm00001d021667*	Probable xyloglucan endotransglucosylase/hydrolase protein 8	−1.07	1.86 × 10^−20^

## Data Availability

Data are contained within the article and Supplementary Materials.

## Supplementary Materials

The following supporting information can be downloaded at: https://www.mdpi.com/article/10.3390/ijms252212437/s1.
