# Supplementary material for: Comparative Analysis of Mesocotyl Elongation Ability among Maize Inbred Lines
Source: Int J Mol Sci. 2024 Nov 19;25(22):12437. doi: 10.3390/ijms252212437 (PMC11595070; doi:10.3390/ijms252212437)
Supplement: Supplementary file 1 [file ijms-25-12437-s001.zip › ijms-3295181-supplementary.pdf]

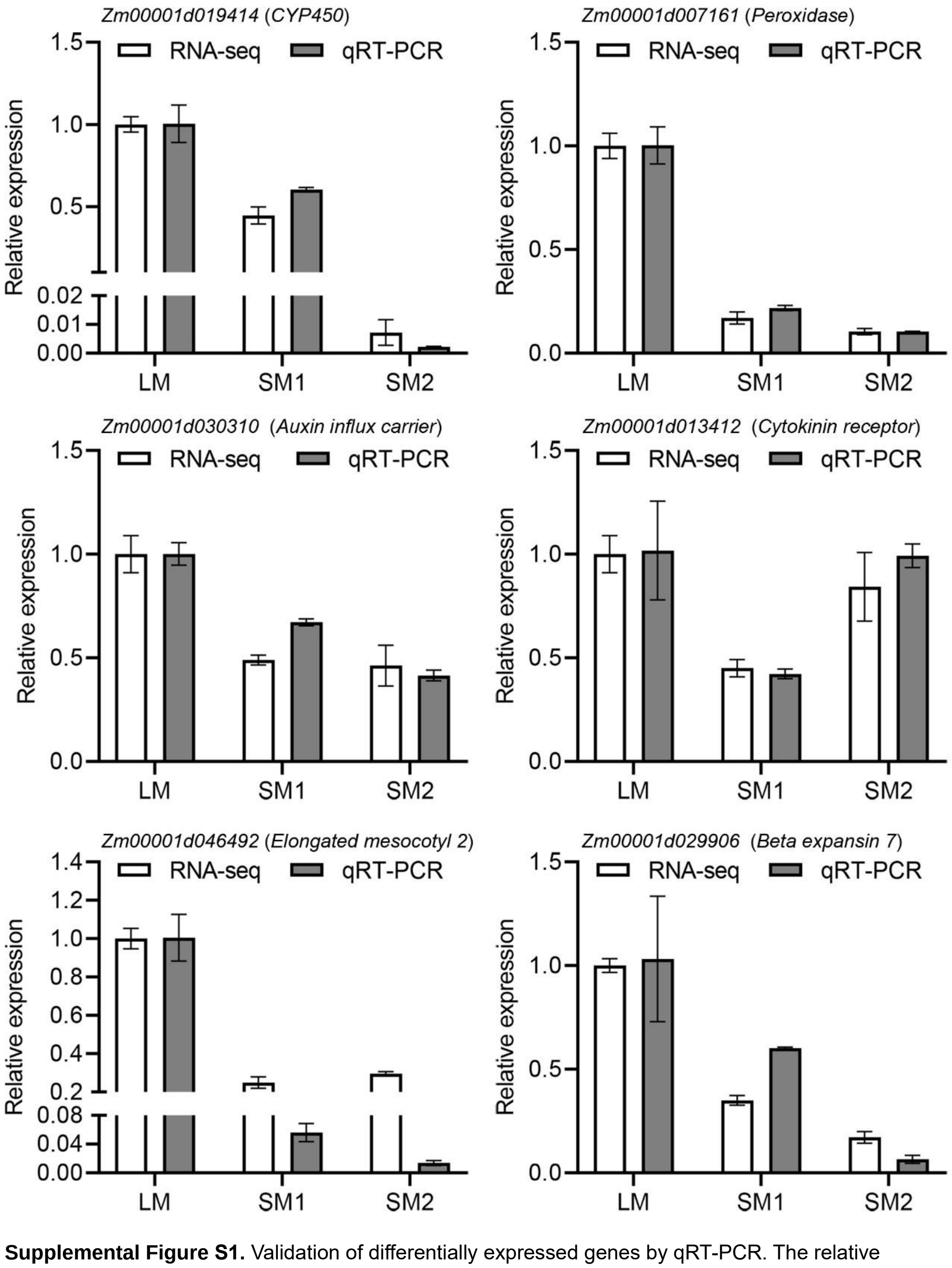

Supplemental Figure S1. Validation of differentially expressed genes by qRT-PCR. The relative expression level of each gene was expressed as a white bar in the RNA-seq data and a gray bar in the qRT-PCR data. The maize *Actin* gene was used as an internal control to normalize the expression data. Error bars represent the standard deviation for three replicates.

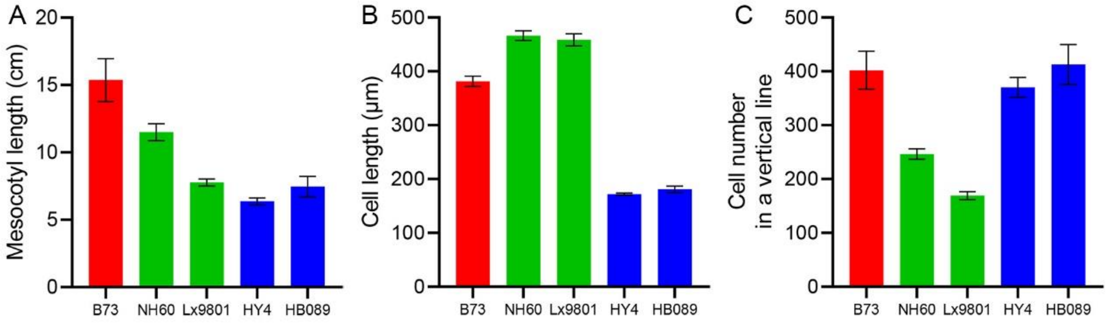

Supplemental Figure S2. Phenotypes of the extra inbred lines in Figure 6. (A) Mesocotyl length; (B) Mesocotyl cell length; (C) Mesocotyl cell number in a vertical line. Red bars represent the maize inbred lines with long mesocotyl. Green bars represent the maize inbred lines with long cells and few cell numbers in mesocotyl. Blue bars represent the maize inbred lines with short cells and many cell numbers in mesocotyl. Error bars display the standard deviation for three replicates.

Supplemental Table S1. Summary of RNA-sequencing data.

Sample	Raw Reads	Clean reads	Total mapped	Uniquely mapped	Multiple mapped
LM_1	63738838	62671164	56583549(90.29%)	55399471(88.4%)	1184078(1.89%)
LM _2	66964866	64474126	58280234(90.39%)	56999985(88.41%)	1280249(1.99%)
LM _3	62471134	61762392	55994840(90.66%)	54801302(88.73%)	1193538(1.93%)
SM1_1	65658740	64672526	57020892(88.17%)	55766062(86.23%)	1254830(1.94%)
SM1_2	61706896	60745864	53708619(88.42%)	52505173(86.43%)	1203446(1.98%)
SM1_3	62109206	61290336	55120160(89.93%)	53838490(87.84%)	1281670(2.09%)
SM2_1	61451004	60396532	52805220(87.43%)	51582111(85.41%)	1223109(2.03%)
SM2_2	62363084	57423594	49242488(85.75%)	48093784(83.75%)	1148704(2.00%)
SM2_3	64080518	59328388	51246721(86.38%)	50121191(84.48%)	1125530(1.90%)

Supplemental Table S2. Primers used for qRT-PCR.

Primer	Sequence (5'to 3')
Actin-F	GCTACGAGATGCCTGATGGTC
Actin-R	CCCCCACTGAGGACAACG
Zm00001d019414-F	AGGAGCAGGCGGATGGTTATG
Zm00001d019414-R	TTCGTCCACCCTTGGTTTGTTTC
Zm00001d007161-F	ACATCAACCCCGCCTTCGCCAT
Zm00001d007161-R	AAAGCGCTGGTGAAGGCTGC
Zm00001d030310-F	CCACCTACACCGCCTGGTACA
Zm00001d030310-R	ATGGCCTTGAACTTCTGAGGAC
Zm00001d013412-F	TTCTGATGCCAAGGAGGAGGAT
Zm00001d013412-R	GGGGTTGCCAAGAGGAACTTCT
Zm00001d046492-F	GGAACAGGAGCCTGTCTGTTTC
Zm00001d046492-R	GTGTGCAGCTTCATCGCGACCT
Zm00001d029906-F	ACAACCTTGTCCTCCACAGTAGT
Zm00001d029906-R	GAGAACGGCGGCAGATTCAC
